# Traditional Uses, Pharmacological Efficacy, and Phytochemistry of *Moringa peregrina* (Forssk.) Fiori. —A Review

**DOI:** 10.3389/fphar.2018.00465

**Published:** 2018-05-11

**Authors:** Annadurai Senthilkumar, Noushad Karuvantevida, Luca Rastrelli, Shyam S. Kurup, Abdul J. Cheruth

**Affiliations:** ^1^Department of Arid Land Agriculture, College of Food and Agriculture, United Arab Emirates University, Al Ain, United Arab Emirates; ^2^Department of Basic Medical Sciences, Mohammed Bin Rashid University of Medicine and Health Sciences, Dubai, United Arab Emirates; ^3^Dipartimento di Farmacia, University of Salerno, Fisciano, Italy

**Keywords:** Moringaceae, medicinal plant, *Moringa peregrina*, traditional uses, pharmacology, phytochemistry

## Abstract

*Moringa* is a sole genus of Moringaceae family with 13 species distributed in the tropical and sub-tropical regions. Among them, *Moringa peregrina* is one of the species which has wide range of traditional, nutritional, industrial, and medicinal values. The plant parts are used in folk medicine for many human health care purposes including diabetes, wound healing, disinfectant, fever, constipation, muscle pains, slimness, burns, labor pain, hypertension, malaria, stomach disorder, asthma, skin problems, and to expel a retained placenta. In addition to medicinal value, *M. peregrina* has cultural, spiritual, and religious connections with the native people of Arabian Peninsula. *M. peregrina* plant parts were tested for many pharmacological activities *viz*, antioxidant, anti-microbial, anti-diabetic, anti-spasmodic, hypertension, hepatotoxicity, lipid lowering activity, anti-inflammatory, anti-cancer, and memory disorders. Few active molecules belong to the class isothiocyanate, flavonoid, triterpenoid, phytosterol, polyphenol, and glycoside were also isolated, identified and reported for anti-microbial, anti-oxidant, anthelmintic, anti-mutagenic, neuroprotective, anti-cancer, anti-hypertensive, anti-diabetic, anti-infective, anti-allergic, anti-inflammatory, herbicidal, lipid lowering potential, anti-trypanosomal, and cytotoxic activities. So, the aim of the present review is to provide comprehensive information from recognized sources on the traditional uses, pharmacological efficacy and phytochemistry of the desert medicinal plant, *M. peregrina*. The information provided in this review will be very useful for further studies to develop novel therapeutic drugs.

## Introduction

Plants play a vital role in cultural, social, religious, environmental, and nutritional aspects. Among all other purposes, the use of plants as medicine for human health originated ~60,000 years ago in the mid-Paleolithic age (Solecki, 1975). To date, 391,000 vascular plants had been identified (Willis and Bachman, 2016). Of these, only about 6% of plants were screened for their biological activity and 15% for their phytoconstituents (Verpoorte, 2000). Among those, *Moringa* is one of the most important genuses with outstanding economic importance. This genus is potentially used in traditional medicine, pharmacological screening and chemical constituents identification. The genus *Moringa* consist of 13 species viz., *M. arborea, M. borziana, M. concanensis, M. drouhardii, M. hildebrandtii, M. longituba, M. oleifera, M. ovalifolia, M. peregrina, M. pygmaea, M. rivae, M. ruspoliana*, and *M. stenopetala*. The history of *Moringa* dates back to 150 BC. The taxon name *Moringa* was derived from the Tamil word “murunggi” or the Malayalam word “muringa” (Quattrocchi, 2000). Historical proof showed that various civilizations viz., Indian, Greek, and Egyptian were using Moringa for thousands of years for several purposes. They preferred to take the leaves and fruits of *Moringa* in their diet to maintain their skin health and mental fitness. In the warfront, the ancient Maurian warriors of India were fed the leaf extracts of *Moringa* as it was believed that the decoction relieves them from the pain and stress incurred during the war. Moreover, the drink provides added energy in the war field (Jahn, 1996; Fuglie, 2001; Manzoor et al., 2007). Edible oil with pleasant taste (Ben oil) from the seeds of *Moringa* was highly valued by the civilizations of ancient Greek, Roman, and Egyptian for protecting their skin and making perfume. Since the middle and old kingdoms (3000–2000 BC), the ben oil was used by the Egyptians (Miller and Morris, 1988; ICUN, 2005).

**Figure 1:**
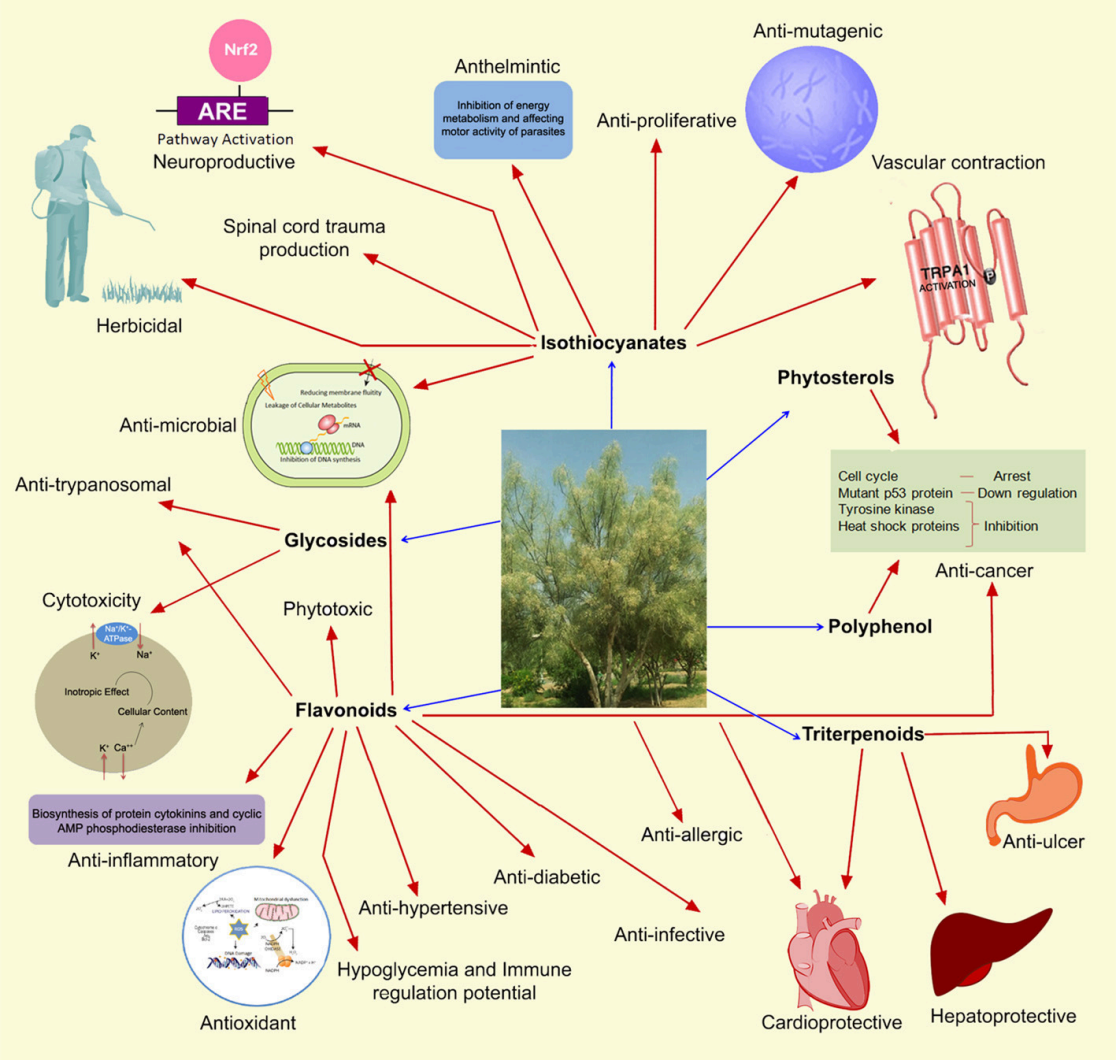
An overview of health applications and salient modes of actions of *M. peregrina*.

The previous studies on the *Moringa* genus were mainly concentrated on *M. oleifera* (Gilani et al., 1994; Pal et al., 1995, 1996; Anwar et al., 2007; Santos et al., 2012; Stohs and Hartman, 2015; Goswami et al., 2016; Leone et al., 2016; Saini et al., 2016; Asensi et al., 2017; Kalappurayil and Joseph, 2017; Mallya et al., 2017; Mangundayao and Yasurin, 2017) since the species is common in Africa and Asia where the common people are search for nutritional foods in an inexpensive way to meet their demand of food sources (Wangcharoen and Gomolanee, 2011). Recently, *M. peregrina* is gaining more attention due to traditional, nutritional, industrial and medicinal values. As this plant has wide range of medicinal uses, it has been screened for various pharmacological activities in the past few decades (Marwah et al., 2007; Soltan and Zaki, 2009; Koheil et al., 2011; Dehshahri et al., 2012; Lalas et al., 2012; Al-Owaisi et al., 2014; Moustafa et al., 2014; Majali et al., 2015; Safaeian et al., 2015; Ullah et al., 2015; Alrayes et al., 2016; El-Awady et al., 2016; Azim et al., 2017; Elabd et al., 2017; Saleh et al., 2017). Few active molecules were also isolated, identified and reported for various pharmacological activities. An overview of health applications and salient modes of actions of phytoconstituents from *M. peregrina* are illustrated in Figure 1. Recently, Robiansyah et al. (2014) reviewed the current status of *M. peregrina* on its nutrient content, medicinal properties, phenotypic and genetic variation and conservation status. But there was no much information about traditional medicinal values, pharmacological activities and phytochemistry of this plant. Therefore, the present review is aimed to summarize the up-to-date information on the traditional uses, pharmacological activities and phytochemistry of *M. peregrina*.

## Botany

*M. peregrina* is a deciduous tree belonging to the family of Moringaceae. It is a fastest growing tree among the other *Moringa* species (Abd El-Wahab, 1995) with 3–10 m height and grayish green bark adapted to high aridity. The leaves are 30–40 cm long, alternate, obovate and deciduous. One of the unique features of *M. peregrina* is the falling of their leaflets when the leaves mature, leaving leaf rachises naked (Robiansyah et al., 2014; Olson et al., 2016). The plant has axillary inflorescence with much branched panicle (18–30 cm long). Flowers are 10–15 mm long, hermaphrodite, zygomorphic, pentamerous, and pinkish white in color with white sepals. A single tree of *M. peregrina* may produce up to 1,000 pods per year and length of the pods may vary from 20 to 40 cm. Each pod contains 8–15 ovoid, un-winged, trigonous seeds (Afsharypuor et al., 2010). Another unique feature of *M. peregrina* is the formation of root tuber in the seedling phase (Munyanziza and Yongabi, 2007).

## Distribution

*M. peregrina* was originated in Arabian Peninsula (Bellostas et al., 2010) and is well-adapted to extreme environmental conditions (Robiansyah et al., 2014). The plant grows in wide geographic range from tropical Africa to East India (Sengupta and Gupta, 1970; Al-Kahtani, 1995; Ghahreman, 2001; Hegazy et al., 2008; Singh et al., 2013). *M. peregrina* is mostly distributed in South and North Hijaz of Saudi Arabia (Migahid, 1978). Jahn et al. (1986) stated that the plant is indigenous as well as cultivated in Sudan. It also grows in Baluchestan, Southeast and Sistan provience of Iran (Ghahreman, 2001). The plant is widely distributed in Yemen, Somalia, Syria, Palestine (Somali et al., 1984), Jordan (Al-Dabbas et al., 2010), and Oman (Al-Owaisi et al., 2014).

## Traditional uses

*Moringa* and its healing potential were documented for the first time around 5000 years ago in the Vedic litrature in India (Patwardhan, 2000). In folk medicine, *M. peregrina* leaf extract is rubbed over skin to treat paralysis and skin rashes (Ghazanfar and Al-Al-Sabahi, 1993). The pod oil is used to treat the convulsions or infantile paralysis in the northern region of Oman (Miller and Morris, 1988). Its seeds are most commonly used to control diabetes in Sultanate of Oman (Al-Kahtani, 1995; Reddy et al., 2015). It is also effectively used for the diabetes related symptoms such as hyperlipidemia and hyperglycemia in the Indian sub continent. The young leaves of *M. peregrina* are used traditionally in folk medicine as antioxidant and wound healing in Arab countries. The bark juice is also used as disinfectant (Marwah et al., 2007) and also to treat fever, headache, constipation, back and muscle pains, slimness, burns and labor pain (Boulos, 2000; Elbatran et al., 2005; Tahany et al., 2010). The leaves are used for wound healing (Nawash and Al-Horani, 2011) and seeds are used for abdominal pain (Van der Vossen and Mkamilo, 2007). The roots and leaves of *M. peregrina* are mixed together with water and used to treat hypertension, malaria, asthma, stomach disorders, diabetes, and to expel a retained placenta (Mekonnen et al., 1999). Traditionally, the oil of this plant is used to treat skin problems such as freckles, itches, and scabies (Al-Dhaheri, 2016).

In addition to their medicinal importance, *M. peregrina* has significant nutritional importance. The young leaves of *M. peregrina* can be used as a vegetable (Al-Dhaheri, 2016). The immature seeds are eaten in India and mature seeds are consumed either roasted or fried in Malawi (FAO, 1988; Elbatran et al., 2005; Afsharypuor et al., 2010). In traditional herbal medication, the seeds of the plant are mixed with other herbs and used as food for anti-malnutrition (MPCP, 2006). In addition, *M. peregrina* is one of the important native trees in the UAE as it has cultural, spiritual, and religious connections. Locally, the leaves of the plant are used to flavor the meat during smoked meat (tanour) preparation. This traditional practice is still followed by the native people of the UAE (Al-Dhaheri, 2016).

## Biological activities

*M. peregrina* parts were tested for broad range of pharmacological activities *viz*, antioxidant, antimicrobial, anti-diabetic, anti-spasmodic, hypertension, hepatotoxicity, lipid lowering activity, anti-inflammatory, anticancer, and memory disorders (Table 1).

**Table 1:** Pharmacological activities of various extracts of *M. peregrina*.

**Plant part used**	**Name of the extract**	**Pharmacological activity**	**Mode of study**	**References**
Bark	Ethanol and aqueous	Antioxidant	*in vitro*	Marwah et al., 2007
Seeds	Hydro-alcoholic	Antidiabetic	*in vivo*	El-Haddad et al., 2002
	Hydro-alcoholic	Anti viral	*in vitro*	Soltan and Zaki, 2009
	Ethanol and aqueous	Anti-inflammatory	*in vivo*	Koheil et al., 2011
	Ethanol and aqueous	Antioxidant	*in vitro*	Koheil et al., 2011
	Oil	Antimicrobial	*in vitro*	Lalas et al., 2012
	Hydro-alcoholic	Lipid lowering	*in vivo*	Rouhi-Broujeni et al., 2013
	Ethanol and aqueous	Antidiabetic	*in vivo*	Koheil et al., 2013
	Ethanol	Antimicrobial	*in vitro*	Hajar and Gumgumjee, 2014
	Ethanol	Antibacterial	*in vitro*	Majali et al., 2015
	Hydro-alcoholic	Anti-spasmodic	*in vivo*	Sadraei et al., 2015
	Oil	Hepatotoxicity	*in vivo*	Sliai and Abdel-Rahman, 2016
	Oil	Anticancer	*in vitro*	Elsayed et al., 2016
	Aqueous extract	Antibacterial	*in vitro*	Saleh et al., 2017
	Oil	Hepatotoxicity	*in vivo*	Elabd et al., 2017
Leaves	Aqueous	Neuroprotective	*in vivo*	Elsaey et al., 2016
	Methanol	Antioxidant	*in vitro*	Dehshahri et al., 2012
	Hexane, chloroform, ethyl acetate and methanol	Antioxidant	*in vitro*	Al-Owaisi et al., 2014
	Methanol	Antioxidant	*in vitro*	Moustafa et al., 2014
	Ethanol	Antimicrobial	*in vitro*	Hajar and Gumgumjee, 2014
	Hydro-alcoholic	Antidiabetic	*in vitro*	Ullah et al., 2015
	Hydro-alcoholic	Anti-spasmodic	*in vivo*	Sadraei et al., 2015
	Methanol and ethanol	Antibacterial	*in vitro*	El-Awady et al., 2015
	Ethanol	Antibacterial	*in vitro*	Majali et al., 2015
	Methanol-aqueous	Antioxidant	*in vivo*	Ullah et al., 2015
	Hydro-alcoholic	Hypertension	*in vivo*	Safaeian et al., 2015
	Methanol and water	Antioxidant	*in vitro*	El-Awady et al., 2015
	Acetone, methanol, ethanol and aqueous	Antibacterial	*in vitro*	Alrayes et al., 2016
	Methanol	Antioxidant	*in vitro*	Juhaimi et al., 2017
	Ethanol	Hepatotoxicity	*in vivo*	Azim et al., 2017
Roots	Ethanol	Antibacterial	*in vitro*	Majali et al., 2015
Aerial parts	Ethanol	Antidiabetic	*in vivo*	Elbatran et al., 2005
Plantlets	Acetone, methanol, ethanol and aqueous	Antibacterial	*in vitro*	Alrayes et al., 2016

### Antioxidant

Reactive oxygen species (ROS) are responsible for the initiation and progression of number of human diseases such as cancer, diabetes mellitus, atherosclerosis, cardiovascular diseases, aging, and cirrhosis (Taniyama and Griendling, 2003). The previous studies indicated that, extracts from plants could prevent or delay the above mentioned diseases owing to their redox properties, which allow them to act as free radical scavengers, reducing agents, and hydrogen donors (Robards et al., 1999; Govindarajan et al., 2005). Along these lines, various extracts of *M. peregrina* were studied for their antioxidant potential. Marwah et al. (2007) studied the antioxidant potential of some plants growing in Sultanate of Oman including *M. peregrina*, which are edible and used for wound healing activity. The aqueous and ethanol extracts of the plant showed a good DPPH scavenging potential with the inhibition up to 87.8% and the IC_50_ value of 7.6 μg/ml. The total antioxidant potential as gallic acid equivalents of ethanol extracts of *M. peregrina* was 814 mg/g. Though, DPPH scavenging potential assay was widely accepted to determine the antioxidant activity of plant extracts, different test methods should be adopted to confirm the potential of the extracts. This single assay can give only a reductive suggestion, because the crude extracts may contain multiple number of compounds with different functional groups (Sacchetti et al., 2005).

Koheil et al. (2011) studied the antioxidant potential of aqueous and ethanol extracts of *M. peregrina* seeds. Reducing power, chelating effect of ferrous ions, DPPH free radical scavenging potential, superoxide anion scavenging potential, hydrogen peroxide scavenging activity, and hydroxyl radical scavenging ability were investigated to find out the antioxidant ability of both ethanol and aqueous extracts of *M. peregrina*. It was observed that the reducing power of the extracts was proportionally increased when the concentration was increased equally. At a concentration of 20 μg/ml, the ethanol and aqueous extracts of *M. peregrina* showed similar reducing power potential similar to that of positive control α-tocopherol (20 μg/ml). The results of the chelating potential of the extracts showed that the activity increased when the concentration was enhanced and chelating activities of ethanol and aqueous extracts were 60 and 37%, respectively at the dose level of 1.50 mg/ml at 90 mts. However, at 1.0 mg/ml concentration the chelating activity of ethanol extract was nearly equal to the positive control EDTA. The ethanol extract of *M. peregrina* showed the highest DPPH radical scavenging activity at the concentration of 6 mg/ml when compared to control. Whereas, 6 mg/ml concentration of aqueous extract showed free radical scavenging potential which was nearly equal to trolox. Superoxide anion scavenging potential of ethanol and aqueous extracts of *M. peregrina* were studied at different concentrations *viz*. 0.1, 0.5, and 1.0 mg/ml and the activity was compared at the same concentration of BHA, ascorbic acid, and trolox. The results revealed that both the extracts showed good superoxide anion scavenging ability than BHA and nearly equal activity to ascorbic acid and trolox. Hydrogen peroxide radical scavenging potential of both the extracts of *M. peregrina* indicated that the activity was in the manner of concentration dependent. Ethanol and aqueous extracts scavenged 79 and 65% of hydrogen peroxide radicals respectively at a dose of 100 μg/ml. Whereas, at the same concentration, control α-tocopherol, BHA, and BHT scavenged 75, 35, and 28%, respectively. It was observed that, both the extracts showed same hydroxyl radical scavenging potential at the concentration of 20 and 40 μg/ml. On the other hand, 20–40 μg/ml concentration of *M. peregrina* ethanol extract showed good hydroxyl radical scavenging potential than the ascorbic acid.

Methanol extract of *M. peregrina* leaves was studied for DPPH free radical and superoxide anion scavenging potential (Dehshahri et al., 2012). The results revealed that the extract scavenged the DPPH radical and superoxide anion radicals with the IC_50_ values of 8.06 and 47.93 μg/ml, respectively. *In vitro* antioxidant activity of hexane, chloroform, ethyl acetate and methanol extracts of *M. peregrina* leaves was studied through the DPPH and H_2_O_2_ scavenging potential. All the extracts showed dose dependent DPPH scavenging potential with the IC_50_ values of 22.36 (hexane), 17.44 (chloroform), 21.87 (ethyl acetate), and 17.07 μg/mL (methanol). The hexane, chloroform, ethyl acetate, and methanol extracts of *M. peregrina* showed significant H_2_O_2_ potential when compared to control. The highest H_2_O_2_ radical scavenging potential was observed at 100 μg/mL of all extracts (Al-Owaisi et al., 2014). The antioxidant activity of the plant samples may also be influenced by the solvents used (Abrahim et al., 2012). Furthermore, polar paradox and polar antioxidants are more potent in lipophilic media whereas nonpolar antioxidants are more active in the polar media (Ramadan and Moersel, 2006).

Moustafa et al. (2014) studied the antioxidant activity of methanol extract of *M. peregrina* along with 199 other wild and cultivated plants in Egypt. DPPH free radical scavenging assay was used to screen the extracts for preliminary antioxidant potential. The methanol extract of *M. peregrina* showed good antioxidant potential (EC_50_ values of 4.4 μg/mL). Antioxidant activity of hydro-alcoholic extract of *M. peregrina* was reported by Ullah et al. (2015). The extract scavenged the ABTS^∙+^ radical in dose dependent manner and the IC_50_ value was 20.56 μg/mL. Elabd et al. (2017) reported that the seed oil of *M. peregrina* showed DPPH scavenging potential at 172 mMol Trolox equivalent/kg.

In comparative study, antioxidant activity (*viz*. DPPH free radical scavenging potential and total antioxidant capacity) of methanol and aqueous extracts of *M. peregrina* were studied and compared with *M. oleifera* (El-Awady et al., 2016). Among the extracts studied, the methanol extract of *M. peregrina* showed high DPPH scavenging activity (165.49 mg ascorbic acid equivalent g extract) and high reducing power potential (335.89 mg ascorbic acid equivalent/g extract). The leaf extract of *M. peregrina* showed good DPPH free radical scavenging potential in concentration dependent manner. The IC_50_ value of the leaf extract was 7.1 μg/ml. While the positive control, ascorbic acid showed the IC_50_ value of 4.6 μg/ml (Azim et al., 2017). The extract of *M. peregrina* young leaves showed 74.78% of DPPH free radical inhibition (Juhaimi et al., 2017). It is evident that the reduction of ROS might have helped the management of degenerative diseases (Valko et al., 2006).

### Antimicrobial

Globally, infectious diseases are the predominant cause of the loss of life. Currently, synthetic antibiotics are widely used to prevent or cure several infectious diseases. The indiscriminate use of synthetic antibiotics poses a serious threat to humans (Lin et al., 2018) as multidrug resistance is developed among the disease causing microbes. Therefore, scientists are more focused on plant based drugs which are no/least toxic to treat the infectious diseases. Moreover, it may help to overcome the emergence of multidrug resistance problem. Therefore, extracts of *M. peregrina* were studied for antiviral, antibacterial and antifungal activities.

In 1997, Mehdi et al. studied the *in vitro* anti hepatitis B viral activity of ethanol extract of *M. peregrina* together with 18 other plants parts against HepG2.2.15 cell line (Mehdi et al., 1997). The results showed that the extract of *M. peregrina* did not inhibit the cell line and survival rate was 100%. Whereas, Soltan and Zaki (2009) screened 42 Egyptian medicinal plants including *M. peregrina* for their antiviral activity and the authors found that the hydro-alcoholic extract of *M. peregrina* demonstrated antiviral potential against herpes simplex-1 virus at concentrations range between 50 and 100 μg/mL with Rf 104. But, the extract inhibited the host cells growth though the experiment was conducted at the maximum concentration of 100 μg/mL. *M. peregrina* extract was found to be inactive against poliomyelitis-1 and vesicular stomatitis viruses.

Disk diffusion method and the determination of minimum inhibitory concentrations were employed to study the antimicrobial potential of *M. peregrina* seed oil against *Staphylococcus epidermidis, Staphylococcus aureus, Escherichia coli, Pseudomonas aeruginosa, Enterobacter cloacae, Klebsiella pneumoniae, Candida albicans, C. tropicalis*, and *C. glabrata*. The activities were compared with the standard antibiotics. The results indcated that the oil was effective against all the tested microorganisms. *C. glabrata* was observed as a most resistant strain among the bacterial and fungal strains. The MIC values of the above mentioned microorganisms were 3.35, 3.50, 4.95, 4.38, 4.80, 4.30, 5.70, 3.30, and 3.25 mg/ml, respectively (Lalas et al., 2012).

Antimicrobial activity of ethanol extract of leaves, seed coat and endosperm of *M. peregrina* were studied by agar well diffusion assay against bacterial (*Bacillus subtilis, Micrococcus luteus, S. aureus, E. coli, P. aeruginosa*, and *K. pneumonia*) and fungal strains (*Aspergillus flavus, Aspergillus fumigatus, Aspergillus niger*, and *C. albicans*; Hajar and Gumgumjee, 2014). The leaf extract of *M. peregrina* showed good antibacterial activity (*B. subtilis* = 20.0 mm; *M. luteus* = 23.67 mm*; S. aureus* = 27.66 mm; *E. coli* = 19.67 mm; *P. aeruginosa* = 26.67 mm; and *K. pneumonia* = 20.67 mm) followed by seed coat (*B. subtilis* = 18.67 mm; *M. luteus* = 20.33 mm*; S. aureus* = 24.0 mm; *E. coli* = 19.33 mm; *P. aeruginosa* = 20.67 mm; and *K. pneumonia* = 13.33 mm) and endosperm (*M. luteus* = 13.33 mm*; E. coli* = 17.67 mm; and *P. aeruginosa* = 16.33 mm. The ethanol extract of *M. peregrina* endosperm had no activity against *B. subtilis, S. aureus*, and *K. pneumonia*. The ethanolic leaf extract also showed good antifungal activity against the tested fungal strains (Leaf extract—*A. flavus* = 23.33 mm*; A. fumigatus* = 22.67 mm; *A. niger* = 18.67 mm; and *C. albicans* = 24.67 mm: seed coat—*A. flavus* = 21.67 mm*; A. fumigatus* = 22.33 mm; *A. niger* = 17.67 mm and *C. albicans* = 22.67 mm: and endosperm—*A. flavus* = 17.33 mm*; A. fumigatus* = 21.33 mm; *A. niger* = 14.67 mm; and *C. albicans* = 20.67 mm). El-Awady et al. (2015) reported the comparative antibacterial activity of *M. peregrina* and *M. oleifera* leaf extracts. The methanol and ethanol extracts of both the plants showed antibacterial activity against *E. coli, S. aureus, Enterococcus sps, Aeromonas hydrophila* and *P. aeruginosa*. But, when compared to *M. oleifera* leaf extracts, *M. peregrina* had less activity.

The antibacterial activity of ethanol extracts of leaves, roots and seeds of *M. peregrina* extracts were investigated against *E. coli, S. aureus*, and *Klebsiella pneumonia* (Majali et al., 2015). The results on the inhibition of studied bacterial strains were concentration dependent. The root extract showed good antibacterial activity against *E. coli* (18–42 mm), *K. pneumonia* (44–59 mm), and *S. aureus* (34–45 mm) followed by ethanol extract of leaf (*E. coli* = 14–30 mm; *K. pneumonia* = 8–19 mm; and *S. aureus* = 9– 22 mm) and seed extract (*E. coli* = 16–38 mm; *K. pneumonia* = 6–32 mm; and *S. aureus* = 6–18 mm). The minimum inhibitory concentrations values of the *M. peregrina* extracts were 12.0 (*E. coli*), 15.0 (*K. pneumonia*) and 18 mg/ml (*S. aureus*) for leaf extract, 13.0 (*E. coli*), 7.0 (*K. pneumonia*), and 9.0 mg/ml (*S. aureus*) for seed extract and 3 (*E. coli*), 5.0 (*K. pneumonia*), and 2.0 mg/ml (*S. aureus*) for roots extracts.

Different extracts (*viz*. acetone, methanol, ethanol, and aqueous) of both *in vitro* plantlets and field grown samples were studied for their antibacterial activity against *K. oxytoca, Salmonella typhimurium*, Methicillin resistant *S. aureus, K. pneumonia, Proteus vulgaris, Proteus mirabilis, Enterobacter aerogenes, P. aeruginosa, E. coli* O157:H7, *S. aureus, Salmonella paratyphi*, and *E. coli* ATCC 29522. The results revealed that *in vitro* plantlets of *M. peregrina* showed significant antibacterial potential when compared to field grown samples. At a concentration of 40 mg/100 μl, the ethanol extract of *in vitro* plantlets of *M. peregrina* showed the highest zone of inhibition against *S. aureus* (Alrayes et al., 2016). Aqueous extract of *M. peregrina* seeds was investigated for antibacterial activity against clinically isolated multidrug resistant *Salmonella* species (Saleh et al., 2017). The results showed that the extracts exhibited good antibacterial activity against the multidrug resistant *Salmonella* isolates. The minimum inhibitory concentration of the extract ranged between 109.37 and 437.5 mg/mL. These results support the use of *M. peregrina* as disinfectant in the folk medicine and further studies can be focused on the isolation of novel antimicrobial molecules to treat the infections caused by microbes.

### Anti-diabetic

Diabetes mellitus is one of the most common metabolic disorders that resulted in significant morbidity and mortality rate (Deshpande et al., 2008). The chronic hyperglycemia of diabetes is associated with prolonged dysfunction, damage, and failure of different organs particularly kidneys, heart, eyes, and blood vessels. There is an increasing demand for traditionally used medicinal plants to manage the diabetes mellitus and its complications since the use of insulin and oral hypoglycemic agents are associated with side effects (Holman and Turner, 1991; Rao et al., 2001). Furthermore, medicinal plants are inexpensive, easily accessible and less or no toxic. Previously, many medicinal plants including *M. peregrina* were reported for hypoglycemic properties (Ahmed et al., 2010).

El-Haddad et al. (2002) reported the antidiabetic activity of hydroalcoholic extract fraction of *M. peregrina* seeds on streptozotocin induced diabetic rats. The administration of hydroalcoholic extract decreased the blood glucose level significantly at the dose of 200 mg/kg b.w. Also the chloroform and petroleum ether fractions decreased the blood glucose level. Furthermore, the histopathological study indicated that the hepatocytes of chloroform treated rats were non toxicated and regenerated the streptozotocin induced diabetic effect. The antidiabetic effect of aerial parts of *M. peregrina* ethanolic extract on streptozotocin induced diabetic rats was reported by Elbatran et al. (2005). The extract significantly decreased the levels of serum glucose, aspartate aminotransferase, and alanine aminotransferase. Also the administration of ethanolic extract of *M. peregrina* has decreased the serum triglycerides, cholesterol and low density lipoprotein. Whereas, the extract increased the level of high density lipoprotein. In toxicological study, the *M. peregrina* extract increased the respiration rate, general depression, mucous membrane cyanoses, righting reflex loss, convulsion and death. The LD_50_ value of alcoholic extract was 113.4 mg/100 g body weight.

The ethanol and aqueous extracts of *M. peregrina* seeds were studied for their anti-diabetic potential in streptozotocin induced diabetic rats through tissue lipid peroxides and enzymatic antioxidant (Koheil et al., 2013). Both the extracts were treated by oral administration at a dose of 150 mg/kg body weight. The results indicated that the blood glucose levels were reduced in the rats administrated with *M. peregrina* seed extracts and glibenclamide (anti-diabetic drug) when compared to untreated diabetic rats. The results on the levels of thiobarbaturic acid reactive substances, nitric oxide, reduced glutathione and hydroperoxides in liver and kidney of treated rats proved that the administration of aqueous ethanolic extracts of *M. peregrina* and glibenclamide were tend to bring down the nitric oxide and reduced glutathione values near to the normal level. The enzymatic antioxidants such as catalase, superoxide dismutase, glutathione peroxidase, glutathione-S-transferase were significantly low in liver and kidney of diabetic control rats when compared to the diabetic rats administrated with ethanol and aqueous extracts of *M. peregrina* seeds and glibenclamide. The diabetic rats administrated with the extracts of *M. peregrina* and glibenclamide showed a decreased level of glycosylated hemoglobin, increased levels of total hemoglobin and plasma insulin when compared to the diabetic control level.

The hydro-alcoholic extract from the dried leaves of *M. peregrina* showed inhibitory potential against three *in vitro* model enzyme assays *viz*. α-glucosidase, α-amylase, and dipeptidyl peptidase IV (Ullah et al., 2015). The results on pancreatic α-amylase inhibitory activity of *M. peregrina* extract suggested that the enzyme responded to the extract when the concentration was increased. The IC_50_ value of the extract was 1335.89 μg/mL. *M. peregrina* extract demonstrated moderate mammalian intestinal α-Glucosidase enzyme inhibitory potential with the IC_50_ value of 3256.68 μg/mL. Whereas, the extract gradually inhibited the activity of mammalian DPP IV enzyme in a dose dependent manner (IC_50_ value of 1218.12 μg/mL).

### Anti-spasmodic

Antispasmodic drugs are prescribed frequently for numerous gastrointestinal illnesses (N'Guessan et al., 2015). Most of the antispasmodic drugs contain antimuscarinic compounds and calcium channel blockers (Farhadi et al., 2001; Pasricha, 2001) and the consumption of these drugs may associate with unwanted side effects. Medicinal plants which are used in folk and traditional medicine for gastrointestinal disorders have been validated through pharmacological studies for antispasmodic activity (Hajhashemi et al., 2000; Sadraei et al., 2003). The results showed that the investigated medicinal plants recorded significant antispasmodic potential (Cortés et al., 2006; Cechinel-Filho et al., 2007). Similarly, the anti-spasmodic potential of hydroalcoholic extract from the leaves and seeds of *M. peregrina* was studied by Sadraei et al. (2015) by ileum contractions induced by 80 mM KCl, 250 μM of acetylcholine (ACh) and electrical field stimulation (EFS). Both the extracts have an inhibitory potential on ileum contractions. The seeds extract of *M. peregrina* had more potential inhibitory effect of ileum contraction induced by KCl (IC_50_ = 87 ± 18 μg/ml); ACh (IC_50_ = 118 ± 18 μg/ml), and EFS (I IC_50_ = 230 ± 51 μg/ml). Whereas, the leaf extract also showed inhibitory effect of ileum contraction (KCl—IC_50_ = 439 ± 108 μg/ml; ACh—IC_50_ = 365 ± 61 μg/ml; EFS—IC_50_ = 314 ± 92 μg/ml). Further investigation on bio assay guided isolation is required to identify the active molecule which could be an alternative and safer anti-spasmodic molecule for future use.

### Hypertension

Hypertension is a cardio vascular disease and it is one of the leading causes of death worldwide. Various anti-hypertensive drugs have been developed for the treatment of hypertension. But the drugs showed efficacy along with associated side effects (Alamgeer et al., 2017). Investigations on edible and medicinal plants remain important since it has potential benefits (Kalia, 2005). Based on the edible importance as well as the traditional uses, the hydroalcoholic extract of *M. peregrina* was investigated on blood pressure and oxidative status in hypertensive rats induced with dexamethasone. Systolic blood pressure, thymus weight, body weight, plasma hydrogen peroxide concentration, plasma ferric reducing antioxidant power were measured after the treatment. The results of the prevention study proved that the extract of *M. peregrina* prevents the rise of systolic blood pressure at 400 mg/kg dose level. Whereas, the reversal study indicated that *M. peregrina* extract failed to lower the SBP in dexamethasone induced hypertension in rats. The oral administration of *M. peregrina* extract had no significant effect on the loss of thymus weight and also the extract was botched to prevent the body weight changes. In contrast, Rouhi-Broujeni et al. (2013) reported that the hydroalcoholic extract from the seeds of *M. peregrina* decreased the mean body weight. In the prevention study, treatment with 200 and 400 mg/kg of extract prevented the rise of H_2_O_2_ concentration. Whereas, in the reversal study, a dose of 400 mg/kg *M. peregrina* extract reduced the elevated plasma hydrogen peroxide concentration. In prevention as well as reversal study, the rats administrated with 400 mg/kg of *M. peregrina* extract significantly reduced the plasma ferric reducing antioxidant power (Safaeian et al., 2015). So, the antihypertensive activity might be linked with the availability of antioxidant molecules present in *M. peregrina*. The antioxidant molecules showed significant role in reducing the level of blood pressure (Duarte et al., 2001; Jalili et al., 2006).

### Hepatotoxicity

The liver is an important organ and plays vital functions in the human body by regulating many biochemical pathways (Sharma et al., 1991). Hepatotoxicity caused by certain drugs/antibiotics, chemicals, microbial infections, and consumption of alcohol is a major concern. Protection of liver using medicinal plants is the best alternative and many plants were reported for anti-hepatotoxicity effect. The seed oil of *M. peregrina* was used for its proteceive effect against doxorubicin induced hepatotoxicity in mice. The reduction in caspase-3 immunoreactivity and apoptotic index were noted in *M. peregrina* seed oil treated mice. Seed oil with the dose of 150 mg/kg treatment reduced the liver damage induced by doxorubicin (Sliai and Abdel-Rahman, 2016). The seed oil of *M. peregrina* was studied along with other two *Moringa* species for liver tissue oxidative stress state in high fat diet induced liver damage. Hepatic marker enzymes, low-density lipoprotein cholesterol, high-density lipoprotein cholesterol, total serum cholesterol, triacylglycerol, glucose, lipid peroxidation, antioxidant enzymes such as catalase, superoxide dismutase, and glutathione peroxidase were analyzed after the treatment. The results indicated that glucose, total serum cholesterol, alanine transaminase, aspartate transaminase, and body weights significantly increased in the rats fed with high fat diet. The post administration of seed oil was significantly improved the liver enzymes, lipid profile, and glucose content (Elabd et al., 2017). The hepatoprotective effect of ethanol leaf extract of *M. peregrina* through oral administration showed that the extract significantly decreased the activities of serum hepatic marker enzymes. The effect of *M. peregrina* leaf extract on oxidative stress markers of acetaminophen induced hepataotoxicity showed that the administration of extract was successful in replenishing the reduced glutathione level in the liver, blood, and brain. In the meantime, the superoxide dismutase, catalase, and glutathione peroxidase activities were reduced significantly in the rats intoxicated with acetaminophen. The superoxide dismutase, catalase and glutathione peroxidase activities enhanced in successful way when the *M. peregrina* leaf extract was administrated (Azim et al., 2017). In literature, triterpenoids have been reported as one of the most important anti-hepatotoxic agents. In the past few decades, more than 350 triterpenoids have been reported for hepatoprotective potential (Xu et al., 2018). So, structure-based investigations are advised to isolate a potent hepatoprotective agent from *M. peregrina*.

### Lipid lowering activity

Hyperlipidemia is closely associated with the coronary heart disease. Hence, lipid lowering therapy alone with the management of other risk factors is advised to prevent the cardiovascular diseases (Jessani et al., 2006). Several drugs are available in the market for lipid related disorders. However, maintenance of lipid homeostasis after the treatment and avoidance of it's the side effect is not an easy task (Pahan, 2006). Thus, plants with medicinal importance are the promising source of lipid lowering active molecule. Hydroalcoholic extract of *M. peregrina* seeds were studied for lipid lowering activity on hyperlipidemic rats (Rouhi-Broujeni et al., 2013) by determining the profile of serum lipid, malondyaldehide, level of thiol, antioxidant capacity, cardiopulmonary resuscitation, ferritin and atherogenic index. The results revealed that the extract of *M. peregrina* significantly reduced the lipid levels such as total cholesterol of plasma, level of LDL-C and VLDL and increased the level of HDL-C in hyperlipidemic rats which is comparable with the lipid lowering activity of the control drug, atorvastatin. Also the level of thiol and carbonyl in the rats administrated with *M. peregrina* extract were same as drug treated rats. The high level of antioxidant capacity and a decreased atherogenic index were also observed in the rats treated with *M. peregrina* extract.

### Anti-inflammatory

In many physiological processes, inflammation is an essential part in response to host defense and the damage of tissues. After injury, the wound healing process starts immediately and the processes comprise of three phases *viz*. inflammation, proliferation, and maturation. The first phase provides resistance to the microbial contaminations (Kondo, 2007) and the anti-inflammatory activity is essential to minimize the healing period (Shimizu et al., 2000). Ethanol and aqueous extracts of *M. peregrina* were studied for anti-inflammatory potential using fresh egg albumin induced inflammation (oedema) in rats (Koheil et al., 2011). The results revealed that the aqueous and ethanol extracts significantly reduced the acute inflammation induced by fresh egg albumin. At a dose level of 300 mg/kg, aqueous and ethanol extracts reduced the inflammation by 72.96 and 81.01%, respectively at the third hour after the oedema was induced. Whereas, the control drug diclofenac at the dose level of 100 mg/kg reduced the inflammation by 100% at the third hour.

### Anti-cancer

Resistance in cancer therapy is a serious issue and it remains as a major cause of death (Batist et al., 2011). The resistance can be developed through various biological mechanisms including reduced drug uptake, increased drug efflux and cellular pathway changes (Tan et al., 2016). It is well-known that plant molecules can be an alternative to the synthetic anticancer drugs to overcome its resistance. Globally, more than 3000 plants have been studied for anticancer properties (Solowey et al., 2014) including *M. peregrina*. *In vitro* anti-cancer properties of seed oil of *M. peregrina* was studied on various cell lines such as MCF-7 (breast cancer cell line), HepG2 (liver cancer cell line), CACO-2 (colon cancer cell line), HeLa (cervical cancer cell line), and L929 (mouse fibroblasts). A significant cytotoxic potential was observed against all the cell lines tested and activity was dose dependent manner. One milligram of the seed oil showed the highest cytotoxic potential against the tested cell lines. Cell viability decreased to 24.65, 24.18, 42.51, 46.57, and 32.11% and the IC_50_ values of the oil were 366.3, 604.3, 850.9, 721.7, and 935.8 μg/mL for HeLa, HepG2, MCF-7, CACO-2, and L929 cell lines, respectively (Elsayed et al., 2016). Based on these results, extensive investigation on the isolation of anticancer molecule is recommended. It could help to overcome the resistance issue as well the lowering the treatment cost.

### Memory disorders

Age related neurodegenerative diseases namely Parkinson's, Huntington's, and Alzheimer's diseases are increased among the human population (Aruoma et al., 2003). Recently, investigations are ongoing to develop new strategies to reduce the disease progression since there is no effective cure for above mentioned disorders (Abushouk et al., 2017). Recent studies indicate that medicinal plants showed good neuroprotection (de Rus Jacquet et al., 2017; Zhang et al., 2017). The neuroprotective effect of aqueous extract from the leaves of *M. peregrina* was investigated and reported by studying the learning capacity and memory in mice (Elsaey et al., 2016). Four doses of the extract were administrated and memory test was performed at two different Zeitgeber times (3:00-rest phase and 15:00-active phase). Insulin was administrated intranasal were treated as positive control. The results on the memory performance showed that intranasal administration of the extract improved the functions of memory close to the positive control insulin. The subchronic administration of the extract at the dose of 25 mg/kg showed significant differences at the Zeitgeber time 3:00 on memory and learning. Whereas, subchronic administration had no significant difference on memory and learning at the Zeitgeber time 15:00. Also it was observed that, in acute administration, there was no locomotor activity observed after intranasal administration of single dose of *M. peregrina* extract neither with any dose nor at both Zeitgeber times. Also there was no significant difference on locomotor activity in sub-chronic administration of the extract. Based on the results, it was concluded that the aqueous extract of *M. peregrina* enhanced the memory function of scopolamine induced amnesia in mice.

## Preliminary phytochemical screening

The desirable therapeutic effects of plant extracts may typically result from the combination of two or more compounds. So, the preliminary phytochemical quantification is essential to correlate the biological activity and it may also help to conduct further studies to discover the particular classes of secondary metabolites. Ullah et al. (2015) screened the hydro alcoholic extract from the dried leaves of *M. peregrina* for its phytochemical compounds. The preliminary quantification analyses showed the presence of major classes of compounds such as alkaloids, tannins, phenolics, and saponins at different concentrations. These phytochemicals are considered to possess extensive range of biological activities (Ramawat et al., 2009). Saponin was recorded in high concentration in the hydroalcoholic extract of *M. peregrina* when compared to other compounds. Dehshahri et al. (2012) reported the presence of flavonoid glycoside (rutin) in the air dried methanol extract of *M. peregrina*. But the authors were abortive to provide the detailed spectral and structure information.

A study was made to quantify the total phenol and flavonoid contents in hexane, chloroform, ethyl acetate, and methanol extracts of *M. peregrina* leaves. The total content of phenols in chloroform, ethyl acetate and methanol were 75.53, 81.26, and 94.56 GAE/g of dry extract, respectively. The results on the total content of flavonoids revealed that 6.55, 8.39, and 20.81 mg of QE/g were present in chloroform, ethyl acetate, and methanol extracts of *M. peregrina* leaves, respectively. On the other hand, phenol and flavonoid contents were not detected in the hexane extract of *M. peregrina*. Also hexane, chloroform, ethyl acetate, and methanol extracts of *M. peregrina* leaves were analyzed by GC-MS to identify the presence of chemical compounds. A total of 32 compounds were identified in all the extracts and all the major chemical compounds namely ethanone, 1-cyclohexyl- (27.26%), pentacosane (17.11%), hexacosane (16.57%), tetracosane (15.45%), heptacosane (13.02), tricosane (11.79%), octacosane (9.10%), cyclopentanol, 1-methyl (8.08%), and 2-heptanone, 3-methyl (7.36%) were identified in hexane extract of *M. peregrina* leaf extracts except p-xylene (10.67%) which was identified in methanol extract (Al-Owaisi et al., 2014).

Safaeian et al. (2015) quantified the total phenol content of dried leaves of *M. peregrina* by Folin-ciocalteu method. The results showed that the dried leaves of *M. peregrina* had 2.3 mg TAE/g of total phenol content. The bark and seed oils of *M. peregrina* contain 454 and 12.6 mg/kg of total phenol content, respectively (Marwah et al., 2007; Elabd et al., 2017). The quantification of total phenol and flavonoid contents of aqueous and methanol extracts of *M. peregrina* were reported by El-Awady et al. (2016) Methanol extract showed a higher amount of total phenol content (137.53 mg gallic acid equivalent/g extract) when compared to aqueous extract (92.26 mg gallic acid equivalent/g extract). The methanol extract also showed high amount of total flavonoid content (33.40 mg quercetin equivalent/g extract) compared to aqueous extract (9.59 mg quercetin equivalent/g extract) of *M. peregrina*.

Saleh et al. (2017) reported the presence of oleic acid-3 hydroxy propyl ester in oily aqueous extract of *M. peregrina* seeds. Though, the authors claimed to successfully purify the compound by GLC and TLC techniques and identified by IR, NMR, and GC-MS, the structure details were not provided. Total flavonoid and phenol content of young leaves of *M. peregrina* were quantified by Juhaimi et al. (2017). The extracts had 35.50 mg catechol gDW^−1^ of total flavonoid and 45.90 mg gallic acid gDW^−1^ of total phenolic compounds. Moreover, the phenolic compounds such as gallic acid (0.930 mg/100 g/DW), protocatechuic acid (0.070 mg/100 g/DW), catechin (0.120 mg/100 g/DW), 4-hydroxybenzoic acid (0.740 mg/100 g/DW), caffeic acid (0.250 mg/100 g/DW), syringic acid (0.08 mg/100 g/DW), rutin trihydrate (0.020 mg/100 g/DW), trans p-coumaric acid (0.140 mg/100 g/DW), chlorogenic acid (0.030 mg/100 g/DW), trans-ferulic acid (0.19 mg/100 g/DW), fisetin (0.030 mg/100 g/DW), trans-resveratrol (0.11), quercetin (0.020 mg/100 g/DW), trans-cinnamic acid (0.270 mg/100 g/DW), naringenin (0.050 mg/100 g/DW), and isorhamnetin (0.330 mg/100 g/DW) were also deducted in *M. peregrina* extracts.

Azim et al. (2017) reported the presence of flavonoids and phenolic compounds in the leaves of *M. peregrina* by HPLC. The highest concentration of flavonoid, reported in mg/100 g dry extract, was rutin (487.3) followed by naringin (45.43), vitexin (16.52), quercetin (14.32), quercetrin (6.96), apigenin (5.43), rosmarinic (3.67), hesperetin (2.27), kaempferol (1.82), naringenin (1.1) and 7-OH flavone (0.31). Whereas 3-OH-tyrosol was recorded in the highest concentration (1763.74 mg/100 g) among phenolic compounds followed by acid vanillic (485.25), protochatecuic acid (444.43), epicatechin (413.1), pyrogallol (243.14), catechol (165.65), salicylic acid (157.65), chicoric (96.9), chlorogenic acid (93.42), benzoic acid (73.57), caffeine (64.31), ellagic acid (60.82), ferulic acid (52.1), *p*-OH-benzoic (51.72), caffeic acid (51.44), iso-ferulic acid (29.8), 4-amino-benzoic acid (23.1), resveratrol (19.5), gallic acid (15.1), *p*-coumaric acid (12.1), 3,4,5-methoxy-cinnamic acid (10.5), coumarin (8.24), cinnamic acid (6.02), and *p*-coumaric acid (1.96). All values were expressed in mg/100 g dry weight.

### Volatile oil composition of *M. peregrina*

Essential oil obtained from the medicinal and aromatic plants gained attention as a potential source in pharmaceutical and food industry due to its efficacy and safety. The volatile oils of leaf and seed kernel of *M. peregrina* were analyzed by GC and GC-MS for its chemical composition. Isobutyl isothiocyanate was identified as a major chemical constituent in both leaf and seed kernel oil with 88.5 and 94%, respectively. Other chemical compounds in leaf oil were isopropyl isothiocyanate (10.2%), *n*-butyl isothiocyanate (0.4%), and hexadecanoic acid (0.2%). Trace amount of sec-butyl isothiocyanate was also deducted in the leaf volatile oil of *M. peregrina*. In seed kernel oil, 4.9% of isopropyl isothiocyanate, 0.5% of sec-butyl isothiocyanate, and *n*-butyl isothiocyanate were identified. In addition, trace amount of n-tridecane, dihydro-α-curcumene, benzyl isothiocyanate, *n*-pentadecane and hexadecanoic acid were also identified in the volatile oil of seed kernel. These compounds were not present in leaf volatile oil of *M. peregrina* except hexadecanoic acid (Afsharypuor et al., 2010).

Salehi et al. (2014) studied the effect of salinity on volatile oil composition of shoot and root of *M. peregrina*. Different levels of salinity *viz*. 2, 4, 6, 8, 12, and 14 dS/m were used to treat the plants and the oil composition was examined by GC-MS. The results indicated that the salinity levels altered the quantity and composition of volatile oil. In control plants, 1,2-benzenedicarboxylic acid, bis (2-methyl propyl) ether was identified as the major compound (29.02%) in the shoots. But in roots, thiocyanic acid, phenylmethyl ester was the major compound. Benzyl isothiocyanate (29.6%) was identified as the major compound in the root samples of *M. peregrina* in all salinity levels. In shoots of *M. peregrina*, isobutyl isothiocyanate was identified as the major compound at 2 and 4 dS/m. Despite in other salinity levels n-butylisothiocyanate was identified as the major compound.

### Fatty acid composition of *M. peregrina* oil

The seed oil of *M. peregrina* was studied by several researchers for its characteristics and chemical composition, mainly on fatty acid. GC analysis of fatty acid methyl esters of *M. peregrina* seed oil showed the presence of 9 fatty acids *viz.*, myristic acid (trace), palmitic acid (9.3%), lauric acid (2.4%) stearic acid (3.5%), oleic acid (78.0%), linoleic acid (0.6%), linolenic acid (1.6%), arachidic acid (1.8%), and behenic acid (2.6%). Among the fatty acids detected, 84.7% were unsaturated fatty acids and 14.7% were saturated fatty acids (Somali et al., 1984). Gas-liquid Chromatography analysis of aerial parts of *M. peregrina* revealed the presence of 13 fatty acids. Palmitic acid was recorded as the major fatty acid (24.68%) followed by myristic acid (16.43%) and linoleic acid (13.60%). Heptadecanoic acid was identified as the lowest (1.38%) fatty acid content. The unsaponifiable matter analysis of *M. peregrina* indicated that β-sitosterol as the major (28.79%) steroidal component (Elbatran et al., 2005).

In 1998, Tsaknis reported the full characterization and chemical composition of *M. peregria* seed oil collected from the Kondom of Saudi Arabia (Tsaknis, 1998). The degummed oil of *M. peregria* had ten fatty acids namely capric acid (0.08%), myristic acid (0.10%), palmitic acid (8.90%), stearic acid (3.82%), oleic acid (70.52%), linoleic acid (0.62%), arachidic acid (1.94%), paullinic acid (1.50%), behenic acid (2.36%), and erucic acid (0.49%). Similar results were obtained with the oil of the seeds collected from Wadi Fenan, Jordan (Al-Dabbas et al., 2010). The analysis of the seed oil showed the presence of high amount of unsaturated fatty acids (83.5%) than saturated fatty acids (16.53%). Among that oleic acid was identified as the major one (74.81%) followed by palmitic acid (8.95), oleic acid (3.72%), stearic acid (3.08%), behenic acid (2.59%), palmitoleic acid (2.28%), arachidic acid (1.73%), eicosenoic acid (1.62%), lingnoceric acid (0.46%), linoleic acid (0.46%), margaric acid (0.12%), myristic acid (0.08%), margaroleic acid (0.08%), and linolenic acid (0.05%). Furthermore, sterol composition analysis of both the oils showed that β-sitosterol as the major one followed by stigmasterol, campesterol and δ-5-avenasterol.

GC analysis showed that the seed oil of Iranian *M. peregrina* contains 82.6% of unsaturated and 17.2% of saturated fatty acid contents. Oleic acid (77.9%) was found to be the major fatty acid followed by palmitic acid (9.3%), stearic acid (3.5%), behenic acid (2.6%) acid, palmitoleic acid (2.5%), arachidic acid (1.8%), linolenic acid (1.6%), and linoleic acid (0.6%) (Gharibzahedi et al., 2013).

Fatty acid composition analysis of *M. peregrina* seed oil by Al-Rawashdeh et al. (2013) revealed that a total of 13 fatty acids were detected in the oil. Oleic acid (78.79%) was identified as the dominant fatty acid, followed by palmitic acid (8.17%), stearic acid (3.85%), behenic acid (2.60%), arachidoic acid (1.99%), palmitoleic acid (1.87%), gadoleic acid (1.57%), linoleic acid (0.47%), lignoceric acid (0.42%), heptadecanoic acid (0.11%), myristic acid (0.07%), heptadecenoic acid (0.07%), and linolenic acid (0.02%).

Fatty acid composition of *M. peregrina* leaf oil was investigated along with two other *Moringa* species (Al-Juhaimi et al., 2016). A total of 12 fatty acids were detected in the leaf oil and linolenic acid recorded the highest (32.53%). Furthermore, considerable amount of linoleic acid (19.5%), palmitic acid (17.6%), oleic acid (7.14%), and arachidic (4.85%) were also detected by GC analysis. Other fatty acids present in the leaf oil of *M. peregrina* were myristic acid, stearic acid, palmitoleic acid, lignoceric acid, behenic acid, and erucic acid with 2.27, 1.96, 1.42, 1.37, 1.19, and 0.76%, respectively. The fatty acid composition analysis (Elabd et al., 2017) revealed that the seed oil of *M. peregrina* contained palmitic acid (10%), linoleic acid (0.42%), oleic acid (78.33%), linolenic acid (2.75%), stearic acid (4.85%), arachidic acid (1.42%), and behenic acid (2.15%).

## Molecules from *M. peregrina* and its biological activity

Medicinal plants are the dominant resource for wide range of molecule structures which helps for the discovery and development of new therapeutic drugs. The therapeutic value of the medicinal plants completely depends on the presence of phytoconstituents and the major group of bio-active compounds present in the plants such as alkaloids, glycosides, flavonoids, proanthocyanidins, tannins, terpenoids, phenylpropanoids, resins, lignans, furocoumarines, naphthodianthrones, proteins, and peptides (Bernhoft, 2010).

**Figure 2:**
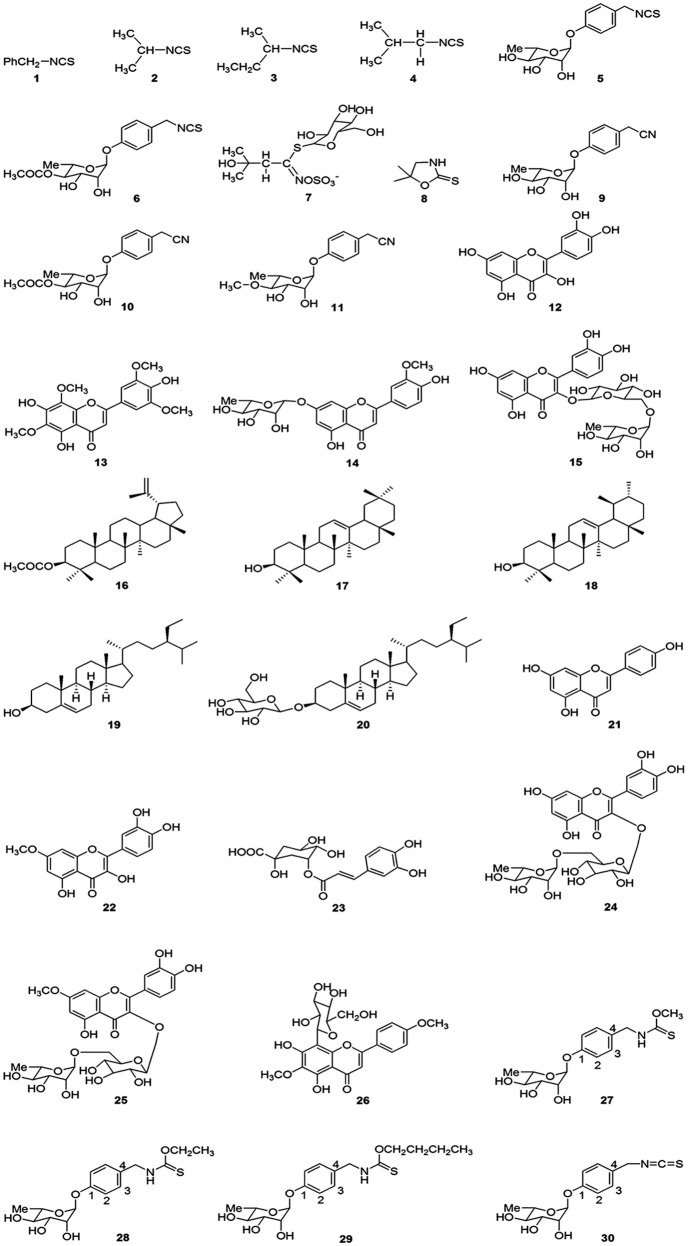
Chemical constituents isolated from various parts of *M. peregrina*.

The literature indicates so far only few studies were made to isolate and identify the phytochemicals from *M. peregrina*. Chemical constituents isolated from *M. peregrina*, plant parts used for isolation, chemical nature and its reported pharmacological activities are illustrated in Table 2 and the molecular structure of the isolated compounds are given in Figure 2. Kær et al. (1979) reported eight isothiocyanates *viz*. benzyl isothiocyanate (**1)**, 2-propyl isothiocyanate (**2**), 2-butyl isothiocyanate (**3**), 2-methylpropyl isothiocyanate (**4**), 4(α-L-rhamnosyloxy) benzyl isothiocyanate (**5**), 4-(4′-*O*-Acetyl-α-L-rhamnosyloxy benzyl isothiocyanate (**6**), glucosinolate (**7**) and 5,5-dimethyl-oxazolidine-2-thione (**8**) from the seeds of *M. peregrina*. But, no biological potential of these compounds were demonstrated. However, benzyl isothiocyanate was reported for anthelmintic (Kermanshai et al., 2001), vascular contraction (Wilson et al., 2002), and antibacterial (Jang et al., 2010) activities. Antimicrobial (Padla et al., 2012), productive effect on spinal cord trauma (Giacoppo et al., 2015) and neuroprotective (Galuppo et al., 2015) activities were also reported for 4-(α-L rhamnosyloxy) benzyl isothiocyanate. Vig et al. (2009) extensively reviewed the antimicrobial, antioxidant, herbicidal, antiproliferative and antimutagenic potential of Glucosinolate. El-Haddad et al. (2002) isolated three nitrile glycosides *viz*, niazirin (**9**), niazirinin (**10**) and 4-(4′- *O*-methyl-α-L-rhamnosyloxy benzyl nitrile (**11**) from the seeds of *M. peregrina*. However, the pharmacological potential of niazirin, niazirinin and 4-(4′- *O*-methyl-α-L-rhamnosyloxy benzyl nitrile were not extensively studied. The aerial parts of *M. peregrina* yielded four flavonoid compounds namely quercetin (**12**), 6,8,3′,5′-tetramethoxy apigenin (**13**), chrysoeriol-7-*O*-rhamnoside (**14**), and rutin (**15**) (Elbatran et al., 2005). Wild distribution of flavonoid compounds in plant kingdom were noted in several studies over the past decades and its concentration varied from plant to plant or even in different parts of the same plant (Justesen and Knethsen, 2001; Dinelli et al., 2006). Flavonoids provids significant health care benefits such as anti-oxidant, antimicrobial, anti-inflammatory, anti-allergic, anti-artherogenic, and cardioprotective (Manach et al., 2005). Quercetin isolated from the aerial parts of *M. peregrina* is one of the important bioflavonoids present in many plants and its various biological activities were already proved in many studies which were complied in several review articles (Sultana and Anwar, 2008; Salvamani et al., 2014; David et al., 2016). Rutin has also received more attention due to its pharmacological activities such as anti-microbial, anti-allergic, anti-diabetes, anti-hypertension, and anti-cancer (Sharma et al., 2013; Gullon et al., 2017).

**Table 2:** Name of the chemical constituents isolated from *M. peregrina*, parts used, chemical nature and its reported pharmacological activities.

**Compounds**	**Names**	**Part used**	**Class of the compound**	**Pharmacological activity**	**References**
1	Benzyl isothiocyanate	Seeds	Isothiocyanate	Antibacterial, anthelmintic, and vascular contraction	Kær et al., 1979; Kermanshai et al., 2001; Wilson et al., 2002; Jang et al., 2010
2	2-Propyl isothiocyanate	Seeds	Isothiocyanate	–	Kær et al., 1979
3	2- Butyl isothiocyanate	Seeds	Isothiocyanate	–	Kær et al., 1979
4	2-Methylpropyl isothiocyanate	Seeds	Isothiocyanate	–	Kær et al., 1979
5	4(α-L Rhamnosyloxy) benzyl isothiocyanate	Seeds	Isothiocyanate	Antimicrobial, Productive effect on spinal cord trauma and Neuroprotective	Kær et al., 1979; Padla et al., 2012; Giacoppo et al., 2015; Galuppo et al., 2015
6	4-(4′-*O*-Acetyl-α-L-rhamnosyloxy benzyl isothiocyanate	Seeds	Isothiocyanate	–	Kær et al., 1979
7	Glucosinolate	Seeds	Isothiocyanate	Antimicrobial, antioxidant, herbicidal, antiproliferative and antimutagenic	Kær et al., 1979; Vig et al., 2009
8	5,5-Dimethyl-oxazolidine-2-thione	Seeds	Isothiocyanate	–	Kær et al., 1979
9	Niazirin	Seeds	Glycoside	–	El-Haddad et al., 2002
10	Niazirinin	Seeds	Glycoside	–	
11	4-(4′- *O*-Methyl-α-L-rhamnosyloxy benzyl nitrile	Seeds	Glycoside	–	
12	Quercetin	Aerial parts	Flavonoid	Antioxidant, anticancer, anti-inflammatory, protection of cardiovascular diseases, anti-hypertensive, anti-diabetic, and anti-infective	Elbatran et al., 2005; David et al., 2016
13	6,8,3′,5′-Tetramethoxy apigenin	Aerial parts	Flavonoid	–	Elbatran et al., 2005
14	Chrysoeriol-7-*O*-rhamnoside	Aerial parts	Flavonoid	–	Elbatran et al., 2005
15	Rutin	Aerial parts	Flavonoid	Anti-microbial, anti-allergic, anti-diabetes, anti-hypertension, anti-cancer	Elbatran et al., 2005; Sharma et al., 2013; Gullon et al., 2017
16	Lupeol acetate	Aerial parts	Triterpenoid	Anti-cancer, analgesic, anti-inflammatory, cardioprotective, and hepatoprotective	Andrikopoulos et al., 2003; Preetha et al., 2006; Saleem, 2009; El-Alfy et al., 2011
17	β-amyrin	Aerial parts	Triterpenoid	Anti-cancer, anti-ulcer, anti-microbial, and anti-inflammatory	Carretero et al., 2008; Holanda-Pinto et al., 2008; El-Alfy et al., 2011; Jabeen et al., 2011; Rosas-Acevedo et al., 2011
18	α-amyrin	Aerial parts	Triterpenoid	Anti-cancer; anti-inflammatory	Holanda-Pinto et al., 2008; El-Alfy et al., 2011
19	β -sitosterol	Aerial parts	Phytosterol	Anti-cancer	El-Alfy et al., 2011
20	β -sitosterol-3-*O*-glucoside	Aerial parts	Phytosterol	Anti-cancer	El-Alfy et al., 2011
21	Apigenin	Aerial parts	Flavonoid	Anti-cancer, anti-oxidant, hypotension, lipid-lowering activity, anti-osteoporosis, hypoglycemia and immune regulation potential	El-Alfy et al., 2011; Zhou et al., 2017
22	Rhamnetin	Aerial parts	Flavonoid	Anti-cancer and Anti-inflammatory	El-Alfy et al., 2011; Jnawali et al., 2014
23	Neochlorogenic acid	Aerial parts	Polyphenol	Anti-cancer	El-Alfy et al., 2011
24	Quercetin-3-*O*-rutinoside	Aerial parts	Flavonoid	Anti-cancer; Antimicrobial, cytotoxic, antioxidant, and phytotoxic	Razavi et al., 2009; El-Alfy et al., 2011
25	Rhamnetin-3-*O*-utinoside	Aerial parts	Flavonoid	Anti-cancer and antihyperglycemic	El-Alfy et al., 2011; Merina et al., 2011
26	6-methoxy-acacetin-8-C- β-glucoside	Aerial parts	Flavonoid	Anti-cancer	El-Alfy et al., 2011
27	*O*-Methyl-4-[(α-L-rhamnosyloxy)benzyl] thiocarbamate	Aerial parts	Glycoside	Antitrypanosomal and cytotoxicity	Ayyari et al., 2014
28	*O*-ethyl-4-[(α-L-rhamnosyloxy)benzyl] thiocarbamate	Aerial parts	Glycoside	Antitrypanosomal and cytotoxicity	Ayyari et al., 2014
29	*O*-butyl-4-[(α-L-rhamnosyloxy)benzyl] thiocarbamate	Aerial parts	Glycoside	Antitrypanosomal and cytotoxicity	Ayyari et al., 2014
30	4-(α-L-Rhamnosyloxy) benzyl isothiocyanate	Aerial parts	Glycoside	Antitrypanosomal and cytotoxicity	Ayyari et al., 2014

In 2011, El-Alfy et al. isolated bio-active molecules which include seven flavonoids [quercetin (**12**), chrysoeriol-7-*O*-rhamnoside (**14**), apigenin (**21**), rhamnetin (**22**), Quercetin-3 -*O*-rutinoside (**24**), Rhamnetin-3-*O*-utinoside (**25**) and 6-methoxy-acacetin-8-C- β-glucoside (**26**)], three triterpenoids [lupeol acetate (**16**), β-amyrin (**17**) and α-amyrin (**18**)], two thytosterols [β-sitosterol (**19**) and β-sitosterol-3-*O*-glucoside (**20**)] and one polyphenol [neochlorogenic acid (**23**)]. The isolated compounds were also investigated for anti-cancer activity against breast and colon cancer cell lines and all the compounds found to be good cytotoxic potential on the cell lines tested. Various other pharmacological activities of the above mentioned compounds have been well-documented in the previous studies. For example, lupeol acetate was reported for analgesic, anti-inflammatory, cardioprotective and hepatoprotective potentials (Andrikopoulos et al., 2003; Preetha et al., 2006; Saleem, 2009). β-amyrin and α-amyrin molecules had close structural relationship with lupeol acetate and known for biological activities such as anti-cancer, anti-ulcer, anti-microbial, and anti-inflammatory (Carretero et al., 2008; Holanda-Pinto et al., 2008; El-Alfy et al., 2011; Jabeen et al., 2011; Rosas-Acevedo et al., 2011). Apigenin is a natural flavonoid and has many pharmacological activities (anti-oxidant, hypotension, lipid-lowering activity, anti-osteoporosis, hypoglycemia, and immune regulation potential) which are recently reviewed by Zhou et al. (2017). A study performed by Razavi et al. (2009) indicated that quercetin-3-*O*-rutinoside had antimicrobial, cytotoxic, antioxidant and phytotoxic potential. Rhamnetin-3-O-rutinoside also a flavonoid showed good antihyperglycemic activity on alloxan induced diabetic rats (Merina et al., 2011). Ayyari et al. (2014) isolated three thiocarbamate [*O*-methyl-4-[(α-L-rhamnosyloxy)benzyl] thiocarbamate (**27**), *O*-ethyl-4-[(α-L-rhamnosyloxy)benzyl] thiocarbamate (**28**) and *O*-butyl-4-[(α-L-rhamnosyloxy)benzyl] thiocarbamate (**29**)] and one isothiocyanate [4-(α-L-rhamnosyloxy) benzyl isothiocyanate (**30**)] glycosides from the aerial parts of *M. peregrina*. The isolated compounds were studied for *in vitro* cytotoxicity in rat skeletal myoblasts and anti-trypanosomal efficacy against *Trypanosoma brucei rhodesiense*. The results revealed that thiocarbamate glycosides had moderate *in vitro* effect, whereas, 4-(α-L-rhamnosyloxy) benzyl isothiocyanate showed significant anti-trypanosomal and cytotoxic potential. Many plant molecules have been isolated and reported for biological activities. But, only few compounds were successfully forwarded from the laboratory to clinical trials. This is due to inadequate information on structure characterization and pharmacological efficacy (Kannathasan et al., 2011). Therefore, sufficient studies on the biological and cytotoxic potential of isolated molecules from *M. peregrina* are suggested for their safe clinical use.

## Conclusions

It is well-known that the researchers always try to discover potent bioactive molecules with least cytotoxicity, as the plant based molecules play a vital role in the development of new modern medicines. *M. peregrina* has rich cultural heritage of traditional healing practices among the people of Arabian Peninsula to treat multiple disorders. Available literature demonstrated that *M. peregrina* were tested for pharmacological activities which are related to traditional uses. Also, different classes of active molecules were also reported in the past few decades. In this review, a comprehensive informations about the traditional uses, pharmacological efficacy and isolated bioactive molecules from *M. peregrina* are documented in order to give collective information for future research. In conclusion, the scientific evidences showed that *M. peregrina* is not fully validated for its pharmacological potential. Moreover, most of the studies were mainly focused on seeds and leaves for pharmacological activities. Since the other plant parts have therapeutic properties, future investigations should be done to evaluate wide range of biological activities of tubers, flowers and seeds. In addition, the phytochemical investigations from the *M. peregrina* are also very limited. Only few molecules were isolated, identified and studied for biological activity. More investigations are needed to explore the efficacy of the medicinal plant and extensive investigations should be carried out to find out the active molecules found in this plant. And also to ensure the safety of the isolated compounds in clinical practice, further investigations are needed to understand the mode of action and toxic potential on host cells.

## Author contributions

All authors listed have made a substantial, direct, and intellectual contribution to the work, and approved it for publication.

### Conflict of interest statement

The authors declare that the research was conducted in the absence of any commercial or financial relationships that could be construed as a potential conflict of interest.
